# Lipidomics Profiling and Risk of Coronary Artery Disease in the BioHEART-CT Discovery Cohort

**DOI:** 10.3390/biom13060917

**Published:** 2023-05-31

**Authors:** Dantong Zhu, Stephen T. Vernon, Zac D’Agostino, Jingqin Wu, Corey Giles, Adam S. Chan, Katharine A. Kott, Michael P. Gray, Alireza Gholipour, Owen Tang, Habtamu B. Beyene, Ellis Patrick, Stuart M. Grieve, Peter J. Meikle, Gemma A. Figtree, Jean Y. H. Yang

**Affiliations:** 1 School of Mathematics and Statistics, The University of Sydney, Sydney, NSW 2006, Australiaellis.patrick@sydney.edu.au (E.P.); 2 Kolling Institute of Medical Research, The University of Sydney, Sydney, NSW 2065, Australiakatharine.kott@sydney.edu.au (K.A.K.);; 3 Department of Cardiology, Royal North Shore Hospital, Sydney, NSW 2065, Australia; 4 Baker Heart and Diabetes Institute, Melbourne, VIC 3004, Australia; 5 Charles Perkins Centre, The University of Sydney, Sydney, NSW 2006, Australia; 6 Department of Cardiovascular Research Translation and Implementation, La Trobe University, Melbourne, VIC 3086, Australia

**Keywords:** CAD, traditional risk factor, imaging technology, lipidomics

## Abstract

The current coronary artery disease (CAD) risk scores for predicting future cardiovascular events rely on well-recognized traditional cardiovascular risk factors derived from a population level but often fail individuals, with up to 25% of first-time heart attack patients having no risk factors. Non-invasive imaging technology can directly measure coronary artery plaque burden. With an advanced lipidomic measurement methodology, for the first time, we aim to identify lipidomic biomarkers to enable intervention before cardiovascular events. With 994 participants from BioHEART-CT Discovery Cohort, we collected clinical data and performed high-performance liquid chromatography with mass spectrometry to determine concentrations of 683 plasma lipid species. Statin-naive participants were selected based on subclinical CAD (sCAD) categories as the analytical cohort (*n* = 580), with sCAD+ (*n* = 243) compared to sCAD− (*n* = 337). Through a machine learning approach, we built a lipid risk score (LRS) and compared the performance of the existing Framingham Risk Score (FRS) in predicting sCAD+. We obtained individual classifiability scores and determined Body Mass Index (BMI) as the modifying variable. FRS and LRS models achieved similar areas under the receiver operating characteristic curve (AUC) in predicting the validation cohort. LRS enhanced the prediction of sCAD+ in the healthy-weight group (BMI < 25 kg/m^2^), where FRS performed poorly and identified individuals at risk that FRS missed. Lipid features have strong potential as biomarkers to predict CAD plaque burden and can identify residual risk not captured by traditional risk factors/scores. LRS compliments FRS in prediction and has the most significant benefit in healthy-weight individuals.

## 1. Introduction

Coronary artery disease (CAD) is the leading cause of death globally. In addition, large epidemiological studies have identified hypertension, hyperlipidemia, cigarette smoking, and diabetes mellitus as modifiable risk factors [1]. With age, sex, clinical lipids, blood glucose levels, and HbA1c, current clinical practice can identify individuals at a high risk of cardiovascular events. However, it is common for an individual to develop severe or fatal events without these well-recognized risk factors [2,3,4]. Hence, biomarkers that can predict risk not captured by standard risk factors are needed.

Computed tomography coronary angiography (CTCA) imaging has evolved to provide high-resolution noninvasive imaging of coronary artery plaque [5]. While it is a readily available clinical tool to examine subclinical CAD, it is also a valuable opportunity for researchers, particularly those interested in discovering novel biomarkers trained against coronary plaque features. Until now, we have relied on cardiovascular events to define individuals with CAD and, the lack of a known event, to identify “healthy” controls. However, if we aim to detect the presence of coronary plaque to allow precise preventative management before a potential heart attack, non-invasive quantification and characterization of plaque—or the lack of plaque—provide substantial advantages. BioHEART-CT is a prospective cohort study and biobank of participants with a clinical indication for CTCA, along with detailed demographic, clinical, and CTCA data to accurately quantify the plaque burden [6] for each participant (Trial registration number ACTRN12618001322224). Its primary aim is to discover blood-based biomarkers of coronary atherosclerosis to guide future precision prevention, with an associated improved understanding of mechanisms informing potential new drug targets.

Total cholesterol, low-density lipoprotein (LDL) cholesterol, and high-density lipoprotein (HDL) cholesterol are widely utilized clinically for their well-established role in the pathogenesis of CAD [7]. However, this is only the “tip of the iceberg” regarding the potential characterization of the diverse range of lipid species that make up these particles. Recent advances in mass spectrometry and high-performance liquid chromatography have allowed more comprehensive studies of the plasma lipidome [8], especially for cardiometabolic diseases that present with significantly altered lipid metabolism. For instance, ceramide is found to be significantly associated with the presence of type 2 diabetes [9] and as a mediator of non-alcoholic fatty liver disease [10]. Therefore, we have developed a lipidomic profiling methodology for cardiovascular and metabolic risk assessment, with the ability to measure multiple lipid species using chromatographic separation coupled with tandem mass spectrometry [8].

In this study, we examined, for the first time, the associations between plasma lipid species and subclinical CAD determined by CTCA imaging analysis. Coronary artery calcium (CAC) score was obtained from calcium dichotomization, whereby participants were categorised into individuals that had greater than or equal to 50 percentile CAC score after adjusting for their age and gender (sCAD+) and individuals with no coronary artery calcification (sCAD−). The Lipid Risk Score (LRS) built in the study had similar predictive power to the existing marker FRS. However, our LRS complemented FRS in predicting sCAD+ by targeting different sets of individuals missed by FRS. Notably, LRS enhanced the risk prediction power for sCAD+ in the healthy-weight sub-cohort (BMI < 25 kg/m^2^), where FRS performed poorly. 

## 2. Material and Methods

### 2.1. BioHEART-CT Study

BioHEART-CT is a multicenter, longitudinal, prospective cohort study of patients with suspected CAD, which brings together detailed clinical information with advanced imaging and molecular phenotyping. It is a sub study of BioHEART, registered with the Australian and New Zealand Clinical Trials Registry (ACTRN12618001322224). The study protocol and design have been described in detail previously [6]. In brief, the study included patients aged 18 or older who had been clinically referred for investigation of suspected CAD by CTCA and were willing and able to provide informed consent. Baseline demographic, past medical history, current medications, and significant social history were collected using a structured data collection form at the time of recruitment [6]. Demographics included age, gender, weight, height, and BMI, and medical history covered the self-reported history of hypertension, hypercholesterolemia, diabetes mellitus, or a smoking history of 10 or more packs per year (significant smoking history). In addition to the self-reported history of hypercholesterolemia, participants taking statins at the time of recruitment were also considered hypercholesterolemia. Current smoking was defined as having regularly smoked within the past 12 months. Data was entered into secure, encrypted databases in a de-identified format.

### 2.2. Study Cohort and Clinical Summary

**Figure 1:**
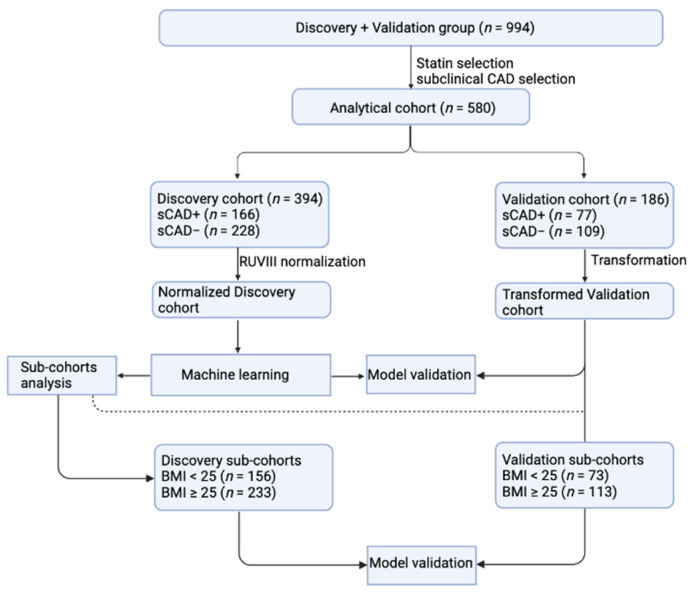
Workflow diagram of statistical analysis. CAD: coronary artery disease; BMI: body mass index.

Our study design consisted of a combined (discovery and validation) group of 994 samples from the BioHEART-CT Discovery cohort, according to the recruitment sequence (Figure 1). CAC score derived from CT imaging analysis is a well-established parameter in evaluating cardiovascular disease severity [11,12]. The analytical cohort (*n* = 580) had been selected to maximize the signal and clinical relevance, where we excluded statin-taking individuals and considered the remaining individuals that have greater than or equal to the 50 percentile of CAC for their age and gender [9] as having subclinical CAD (sCAD+, *n* = 243) and individuals with no coronary artery calcification as no subclinical CAD (sCAD−, *n* = 337). According to the discovery and validation setting, the analytical cohort was then split to generate the discovery cohort (*n* = 394, comprising 166 sCAD+ samples and 228 sCAD− samples) and the validation cohort (*n* = 186; with 77 sCAD+ samples and 109 sCAD− samples). Table 1 summarises baseline demographic and clinical data, including risk factors and regular medication according to sub-cohorts. Categorical variables including gender, hypertension, diabetes mellitus, hypercholesterolemia, current smoking status, Standard Modifiable cardiovascular Risk Factors (SMuRF) [2], significant family history, and current medications (oral anticoagulant, antiplatelet, statin, beta-blocker, angiotensin-converting enzyme (ACE) or angiotensin receptor blockers (ARB), calcium channel blocker and diuretics) are presented in the format of number and percentage. Continuous variables, including age, body mass index, CAC, Gensini, and Soft Plaque Score (SPS) [13], are concluded in mean and standard deviation. The calculation was performed by using the *gtsummary* (v.1.5.2) package. 

### 2.3. Experimental Design, Sample Preparation, and Lipid Extraction

For each individual during recruitment, we ensured the individual had technically adequate CTCAs and sufficient stored blood samples for the planned biomarker discovery platforms. We did not have a cardiac stent in situ or a prior coronary artery bypass graft surgery history. Venous blood (20–30 mL) was collected, processed and stored at −80 °C for later analysis. Before lipid extraction, the sample sets were randomized, with pooled plasma quality control samples interspersed every ten samples and blanks every 20 samples. After adding non-endogenous (e.g., deuterated) lipid internal standards corresponding to the significant lipid categories and classes/sub-classes of interest, lipids were extracted by our single-phase butanol/methanol method that provides high recovery of all lipid classes [14].

### 2.4. Lipidome Quantification and Processing

Plasma (10 μL) was subjected to reverse-phase high-performance liquid chromatography (HPLC) coupled to electrospray ionization-tandem tandem mass spectrometry (ESI-MS/MS) to quantify lipid classes and species according to the previously described method [8]. These species have previously been shown to be associated with coronary artery disease. We calculated the concentration of each lipid species by comparing the peak area to the corresponding deuterated and/or non-physiological lipid species as internal standards. For each sample, we obtained concentrations of 683 lipid species and performed log transformation on concentrations for the subsequent analysis.

### 2.5. Remove Unwanted Variation (RUV) Normalization for Discovery Data-Set

Participants were recruited in consecutive order, and blood samples were stored in batches for the convenience of the study. To detect potential batch effects, we performed a principal component analysis (PCA) based on all the 683 lipid species. We checked principal components against the batches, followed by a RUVIII normalization [13] using pseudo-replicates to correct batch effects. We quantified the association between the principal components (PC) and the wanted and unwanted variation. We determined the coefficient of determination (*R*^2^) by applying a linear regression model for each PC, with batch (unwanted variation), gender and age (wanted variation) variables, respectively. 

We then conducted a RUVIII normalization [15] incorporating the idea of identifying pseudo-replicates computationally [16] to remove unwanted variances and estimate alpha. Briefly, we considered samples within roughly known biologically homogenous sub-class of samples as pseudo-replicates. We applied the Agglomerative Hierarchical Clustering method to achieve 80 pseudo-replicates based on age, gender, BMI, hypertension, current smoking status, diabetes mellitus, and hypercholesterolemia status of the samples. Pooled QC samples, technical QC samples, blank samples, and NIST1950 samples are treated as four pseudo-replicates to facilitate normalization. For the control lipids, we consider lipids significantly associated with batch effects and exclude lipids significantly associated with age and gender. We consider k = 8 factors of unwanted variation. For the validation cohort, we first standardized the data with means centered to zero to estimate the unobserved covariate (W) matrix using the coefficients of the unobserved covariates (alpha) derived from the discovery set. Next, we adjusted the data by subtracting the product of W and alpha from the standardized data and adding the feature-wise means as the final adjusted validation data. The adjusted discovery and validation data were used in the subsequent analyses. 

### 2.6. Association Study—Logistic Regression

For the association test between the binary categories of CAD and lipid features, we fit logistic regression models for each of the lipid species (*n* = 683) with sCAD−/sCAD+ as the dependent variable, using the post-normalization data (discovery and validation cohort combined). For each lipid species, logistic regression was constructed utilising the lipid concentration as the only independent variable and a model including additional clinical variables, including BMI, hypertension, current smoking status, diabetes mellitus, and hypercholesterolemia. We consider each lipid species with the log-transformed concentration of lipid species as the independent variable. The level of associations was quantified as odds ratios with 95% confidence intervals. Significantly associated lipid measures are selected by controlling for a 5% Benjamini-Hochberg false discovery rate (adjusted *p*-values < 0.05). 

### 2.7. Risk Model Building

Via a bootstrapping approach, we selected alpha and the number of informativity-based top lipid species features that give the highest AUC (Supplementary Figure S1) in predicting sCAD+/sCAD−. The final risk model is constructed using a ridge regression with sCAD−/sCAD+ as the dependent variable and the top 200 lipid species as the independent variables. We fit a logistic regression model for FRS [17] with sCAD−/sCAD+ as the dependent variable, resulting in the FRS model. For the FRS + LRS model, we fit a ridge regression with sCAD−/sCAD+ as the dependent variable and 200 lipid species (from LRS) and FRS as the independent variables. Models were constructed on the discovery cohort only. 

### 2.8. Subcohort Analysis

To identify cohort heterogeneity and the potential to improve prediction performance for sCAD+ that may be apparent in specific clinically relevant subpopulations, we adapted a previous procedure developed in other omics settings and for melanoma [18]. We first estimated the individual classifiability scores (ICS) and identified a modifying clinical variable with the highest association with ICS. That is, depending on the level of the modifying variable, there is a different association between sCAD+ and the lipidomic profile. Following a ridge regression fitted to the discovery cohort using 200 species, individual classifiability (accuracy rate for the individual) was determined as the proportion of correct predictions via a 50-repeated five folds cross-validation strategy, with functions calcCVperformance and performance from the *ClassifyR* package. To investigate the association between individual classifiability and identify candidate modifying variables (informative variables including BMI, hypertension, and hypercholesterolemia), we fit a linear regression model using individual classifiability score as the output variable and candidate variables as the input variables, adjusted for age and gender. BMI showed the least *p*-value in the association analysis (Supplementary Table S1). Hence we chose BMI as the modifying factor. We then divided the cohort at a BMI of 25 kg/m^2^ according to common clinical practice [19]. 

### 2.9. Model Validation and Visualization

For each validation set of cohorts (or sub-cohorts), a Receiver Operator Curve (ROC) was generated based on the risk model. FRS and LRS built from the discovery cohort level were compared to determine the model targets, i.e., individuals that can be precisely predicted by the model. 

## 3. Results

### 3.1. Lipidomics Profiling for the BioHEART-CT Discovery Cohort

This total available study cohort for discovery and validation included 994 samples from the BioHEART-CT discovery study. We excluded individuals taking a statin and then formed two groups for analysis- those with no coronary calcification (sCAD−) and those whose CAC was in the top 50% percentile compared to individuals of the same sex and similar age (sCAD+). The analytical cohort comprises 394 (sCAD+ = 166; sCAD− = 228) individuals and 186 (sCAD+ = 77; sCAD− = 109) individuals from the validation cohort. 

A summary of baseline clinical and demographic data, including risk factors and regular medication for each sub-cohort, is shown in Table 1. Advanced lipidomic measurements were performed using high-performance liquid chromatography with mass spectrometry. The lipidomics profiles were generated for each individual, including clinical data and 683 lipid species used for ensuing analyses.

### 3.2. RUVIII Normalization of Lipidomics Data

We performed a RUVIII normalization using pseudo-replicates to correct batch effects. Before the normalization, the PC5 and PC6 showed the highest *R*^2^ among the first 30 PCs, at 0.081 and 0.168, respectively. The *R*^2^ were both reduced to less than 0.01 after the normalization. Supplementary Figure S2 shows the PCA plot before and after the normalization and suggests mitigation of the batch effect. The adjusted data were used for the subsequent analyses. 

### 3.3. Association Analysis of Lipidomic Data

**Figure 2:**
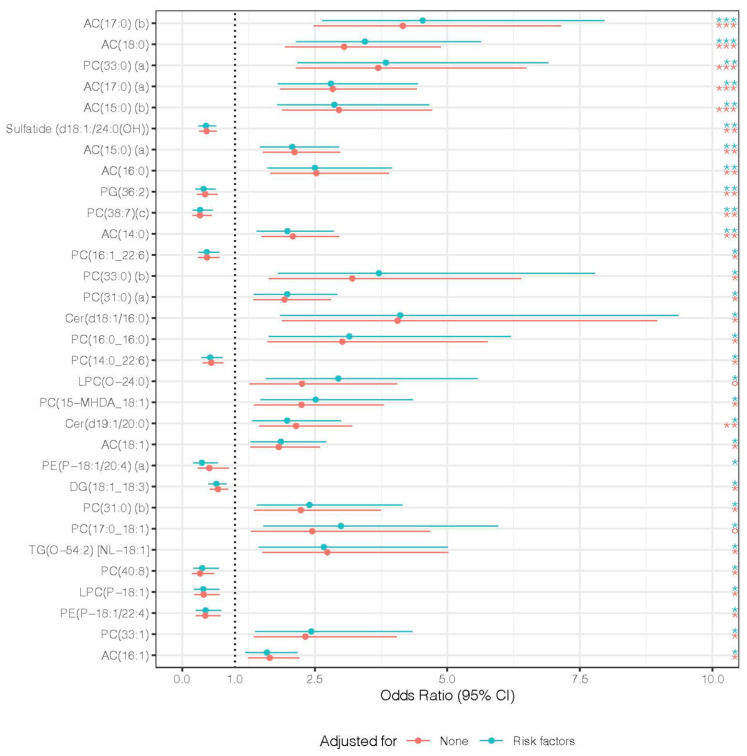
Forest plot of lipid species association results. For each lipid species, a logistic regression model was built with subclinical CAD binary classes sCAD+/sCAD− as the dependent variable and normalized abundance of species as the independent variable, and then adjusted for BMI index, hypertension, current smoking status, diabetes mellitus, hypercholesterolaemia. Odds ratios of lipid species significantly associated with CACS before and after adjustment are shown. The significance was determined by adjusted *p*-values via Benjamini-Hochberg false discovery rate procedure at a cutoff of 0.05, with * for *p* < 0.05, ** for *p* < 0.01, and, *** for *p* < 0.001. Red represents non-adjusted univariate associations, and turquoise represents adjusted associations. The lipid species are arranged by adjusted *p*-values (of adjusted association) in ascending order. The line represents the 95% confidence interval of each odds ratio. Lipid class abbreviations: AC, acylcarnitine; PC, phosphatidylcholine; PG, phosphatidylglycerol; Cer, ceramide; LPC, lysophosphatidylcholine; PE, phosphatidylethanolamine; DG, diacylglycerol; TG, triacylglycerol.

We next studied the association of lipidomic profiles with subclinical CAD, sCAD+/sCAD−. There were 31 lipid species significantly associated with sCAD+ after adjustment for available clinical risk factors (Figure 2). Among the species, LPC(O-24:0) and PC(17:0_18:1) were not significantly associated with subclinical CAD independent of risk factors but were significant after adjustment. Within the 31 species, acylcarnitine and phosphatidylcholine species were the most represented (9 and 12, respectively) as significantly associated with sCAD. 

### 3.4. Comparison between FRS and LRS in Predicting sCAD

**Figure 3:**
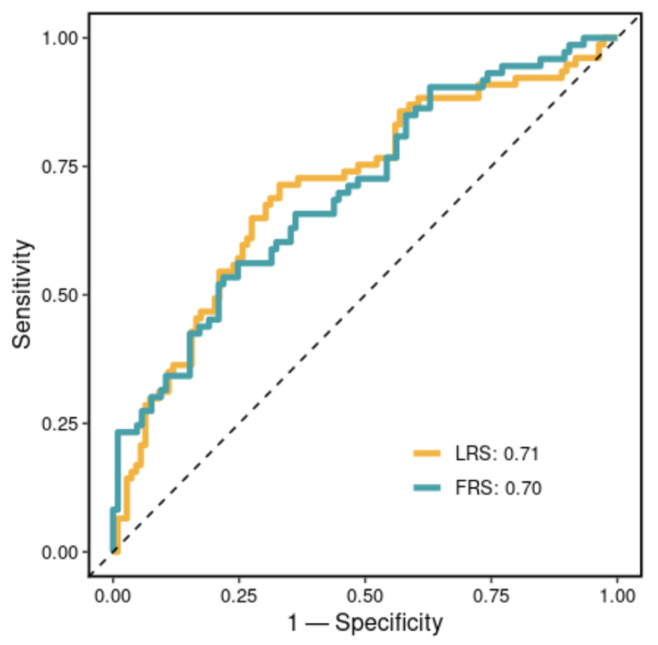
Receiver Operating Characteristic (ROC) curves of risk models in predicting the validation corhot. The lipid Risk Score (LRS) model was fitted and built within the discovery cohort, with subclinical sCAD binary classes sCAD+/sCAD− as the dependent variables and the top 200 selected species features. For the FRS model, a logistic regression was fitted and built within the discovery cohort, with subclinical CAD binary classes sCAD+/sCAD− as the dependent variables and Framingham Risk Score (FRS) as the independent variable. The ROC curves were generated for the prediction of the validation cohort. Area Under Curves (AUC) are shown in the legend.

To identify lipid features with potential prognostic significance for subclinical CAD, we applied a machine learning workflow. We built a (i) lipid risk model (LRS) and (ii) FRS model and then examined the performance of the two models in predicting sCAD+/sCAD−. Figure 3 shows Receiver Operating Characteristic (ROC) curves for the validation cohort predicted by LRS and FRS. LRS had an AUC of 0.71, and FRS had an AUC of 0.7. There were no significant differences between the performance of the two models (*p*-value = 0.84, DeLong’s test Z = 0.20). This suggests both LRS and FRS can predict a large proportion of the subclinical CAD class. After adding lipid species set to FRS, there was no significant improvement in AUC compared to FRS alone. 

### 3.5. Stratification of Cohort Using BMI

**Figure 4:**
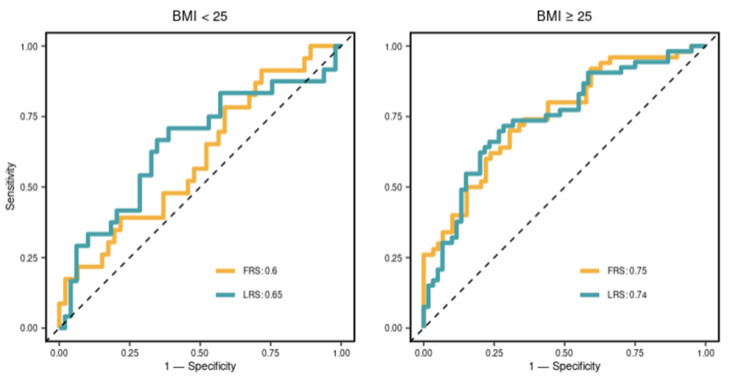
Receiver Operating Characteristic (ROC) curves of risk models in predicting the sub-cohorts. For each sub-cohort, the lipid Risk Score (LRS) and FRS risk model derived from the discovery cohort level were fitted to the validation sub-cohort. The ROC curve was generated in predicting the corresponding validation sub-cohort. Area Under Curves (AUC) are shown in the legend.

To identify cohort heterogeneity, we associated clinical variables with individual classifiability scores. We found that BMI showed the least *p*-value in the linear regression after adjusting for age and gender, leading to our strategy of stratification by BMI. Thus, BMI at 25 kg/m^2^ was selected as the stratification criterion, according to the common practice in the clinic. The stratification resulted in two sub-cohorts: (i) BMI < 25, discovery sub-cohort *n* = 156 and validation sub-cohort *n* = 73; and (ii) BMI ≥ 25, discovery sub-cohort *n* = 235 and validation sub-cohort, *n* = 113. We compared the prognostic utility of two sets of markers, FRS and LRS, in the sub-cohorts. As shown in Figure 4, the performance of LRS was different in two BMI sub-cohorts, achieving AUCs of 0.65 and 0.74, respectively. The relative benefit of the LRS was greatest in the healthy weight individuals where the FRS was seen to perform relatively badly (AUC at 0.6). Indeed, LRS significantly improved the prediction in individuals with a BMI < 25. However, in those with a BMI ≥ 25, FRS and LRS were similarly informative.

### 3.6. The LRS and FRS Model Targets a Different Set of Individuals

**Figure 5:**
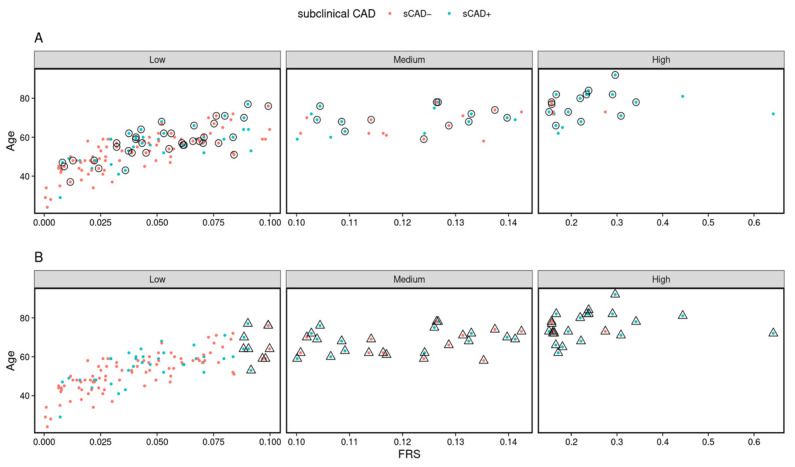
High-risk individuals are predicted by two risk models. Scatterplot comparing age (y-axis) versus the Framingham Risk Score (x-axis) for each individual from the validation cohort. Each point refers to individuals, and the colour highlights they are in the subclinical CAD binary classes, sCAD− (red) or sCAD+ (blue). Panel (**A**) highlights (by a circle) the individuals selected based on LRS, and (**B**) highlights (by triangle) the individuals selected based on the FRS model.

To determine whether our LRS targets the same individuals as the popular FRS model, we compared the risk prediction for each individual between two risk models (Supplementary Figure S3) in two BMI sub-groups. Figure 5 shows the scatter plot of the whole validation cohort predicted based on LRS (Figure 5A) and FRS (Figure 5B), with age and FRS. Both risk models correctly classify most samples (as indicated in Figure 3). However, 31.5% (23 out of 73) samples within sCAD+ were correctly classified by both risk models. In addition, we found that 38 samples within sCAD+ were correctly identified by the FRS model compared to 41 by the LRS. More importantly, around 24.7% (18 out of 73) of samples detected as sCAD+ by LRS were considered sCAD− based on FRS and hence, missed by the FRS model. This indicates that while the AUCs achieved are similar by both models, the two models capture different collections of individuals and demonstrate that the lipid combination features have the potential to predict subclinical CAD residual risk.

## 4. Discussion

For the first time, we investigated the association between subclinical CAD burden and phenotype defined by CT imaging data with an advanced personalized lipidomic dataset involving 683 plasma lipid species. We deployed a machine learning approach to selecting lipid features and building a ridge regression model to establish a new lipid score, LRS, for predicting higher than 50 percentile calcium plaque burden derived from CT imaging analysis (sCAD+). A sub cohort analysis further showed that LRS enhanced the prediction power in the healthy-weight group, indicating the potential of lipid features as biomarkers for identifying the presence of subclinical CAD in individuals where FRS performs poorly. Furthermore, the LRS complemented the prediction of the FRS in evaluating coronary artery plaque burden as a novel strategy to prevent heart attack. 

This study aimed to facilitate the detection of clinically significant but potentially asymptomatic CAD to drive precision prevention strategies with effective therapies for stabilizing plaque, preventing plaque progression, and preventing cardiovascular events. As such, we used non-invasive imaging of coronary artery plaque burden by CAC from CTCA imaging analysis rather than cardiovascular events to inform our models. CACS was derived from a non-contrast CT scan and provided a highly quantifiable measure reflecting atherosclerotic burden. 

Lipids are associated with cardiovascular disease events [20]. In this study, we provided evidence that lipids were also associated with artery plaque burden. Among the significantly associated lipids, acylcarnitine (AC) and phosphatidylcholine (PC) were the two main lipid classes that contain the most amount of lipid species, and these two lipid classes, as well as ceramide (Cer), were reported as biomarkers predicting cardiovascular events and other diseases that are closely related to cardiovascular diseases [21,22]. For instance, ACs are associated with an increased risk of atrial fibrillation [23], and Cers are at higher levels in patients with diabetes [24]. Our results indicated that these three classes played an essential role in the development of CAD. Other lipid classes with a smaller number of significantly associated lipids in this study, e.g., sulfatide and lysophosphatidylcholine, also serve as biomarkers for CAD events [25,26]. Among the lipid classes, lysophosphatidylcholine plays a crucial role in human atherosclerotic plaque inflammation [27]. Hence, we suggested further study to reveal the association of lipid classes with events and calcium plaque burden. 

FRS is a well-established score that takes traditional risk factors to predict CAD events and is extensively used itself, or with minor modifications, in current clinical practice [28]. However, it is well recognized that these scores do not serve all equally. Women and non-white individuals are examples not targeted by the risk score [29,30]. The relatively young and those without those risk factors may still develop CAD and subsequent events without the opportunity to utilize effective preventative medication. One strategy to address this challenge is via sub-cohort analysis. This is not a new concept in medical research, and traditionally, a knowledge-driven strategy is used to partition the data. For example, the most common practice is stratification by gender, as it is well-known as the risk factor for CAD. Instead of a knowledge-driven strategy, our approach here uses a data-driven or computational strategy. This identified BMI as a key modifying variable which we then used to stratify cohorts into sub-cohorts grouped by BMI, which has been extensively utilized in obesity studies [31]. Following the data-driven approach and stratification based on BMI, we consider both FRS and LRS in the prediction. The improvement in the prediction of coronary artery plaque burden is two-fold: (1) in the healthy-weight (BMI < 25 kg/m^2^) group, LRS increases the prediction power, raising AUC from 0.6 to 0.65. This aligns with our task in the BioHEART-CT study to identify patients with the absence of traditional risk factors. (2) In both BMI groups, LRS can identify high-risk samples overlooked by FRS. This will benefit the diagnosis and allow precise management of early CAD.

One of the limitations of the study is the sample size. The LRS model was built using the coronary artery calcification derived from the CTCA image as the outcome. Due to the specificity, a separate and similar cohort with lipidomic assessment is not currently available. To address the issue of reproducibility, we split the cohort into discovery and validation cohorts according to the order of individual recruitment. We treated the validation cohort as a separate cohort for the model validation. 

In conclusion, we utilized lipid features from an advanced lipidomic platform to predict the presence of subclinical CAD, defined as a greater-than-average coronary artery calcium score (sCAD+) adjusted for age and gender. Notably, we selected a subset of plasma lipid species. In addition, we performed sub-cohort analysis as a data-driven strategy to identify populations where the LRS could best add value in detecting early disease. In healthy-weight individuals, our new LRS provided valuable advances over traditional risk scores. Meanwhile, LRS complemented FRS in the prediction of coronary artery plaque burden. These results provide insight into applying lipidomics data in CAD diagnosis, prevention, and treatment.

## Figures and Tables

**Table 1:** Demographic and clinical information of study cohorts.

Characteristic	Whole Cohort	Discovery Cohort		Validation Cohort	
	*n* = 994	sCAD−, *n* = 228	sCAD+, *n* = 166	sCAD−, *n* = 109	sCAD+, *n* = 77
Age-years, mean (SD)	61 (12)	53 (11)	62 (11)	55 (11)	64 (12)
Female, *n* (%)	444 (45%)	121 (53%)	61 (37%)	66 (61%)	24 (31%)
BMI-kg/m^2^, mean (SD)	26.9 (4.8)	26.3 (4.6)	27.4 (5.4)	26.7 (5.1)	27.3 (4.3)
Hypertension, *n* (%)	384 (39%)	48 (21%)	70 (42%)	34 (31%)	35 (45%)
Diabetes Mellitus, *n* (%)	86 (8.7%)	7 (3.1%)	12 (7.2%)	8 (7.3%)	5 (6.5%)
Hypercholesterolaemia, *n* (%)	585 (59%)	76 (33%)	71 (43%)	42 (39%)	37 (48%)
Current Smoking Status, *n* (%)	64 (6.4%)	14 (6.1%)	15 (9.0%)	6 (5.5%)	9 (12%)
SMURFs, *n* (%)					
0	216 (22%)	104 (46%)	34 (20%)	35 (32%)	13 (17%)
1	411 (41%)	93 (41%)	78 (47%)	53 (49%)	40 (52%)
2	273 (27%)	28 (12%)	45 (27%)	18 (17%)	19 (25%)
3	80 (8.0%)	3 (1.3%)	8 (4.8%)	3 (2.8%)	5 (6.5%)
4	14 (1.4%)	0 (0%)	1 (0.6%)		
Significant Family History CAD, *n* (%)	225 (23%)	50 (22%)	38 (23%)	19 (17%)	8 (10%)
Oral anticoagulant, *n* (%)	91 (9.2%)	16 (7.0%)	13 (7.8%)	8 (7.3%)	15 (19%)
Antiplatelet, *n* (%)	176 (18%)	20 (8.8%)	13 (7.8%)	10 (9.2%)	5 (6.5%)
Statin, *n* (%)	331 (33%)	0 (0%)	0 (0%)	0 (0%)	0 (0%)
Beta Blocker, *n* (%)	143 (14%)	20 (8.8%)	24 (14%)	10 (9.2%)	13 (17%)
ACE or ARB, *n* (%)	316 (32%)	32 (14%)	58 (35%)	23 (21%)	28 (36%)
Calcium Channel Blocker, *n* (%)	105 (11%)	9 (3.9%)	24 (14%)	8 (7.3%)	7 (9.1%)
Diuretics, *n* (%)	82 (8.2%)	11 (4.8%)	16 (9.6%)	3 (2.8%)	12 (16%)
Coronary Artery Calcium Score, mean (SD)	211 (614)	0 (0)	287 (461)	3 (30)	404 (645)
Gensini Score, mean (SD)	9 (13)	1 (2)	14 (14)	1 (4)	15 (17)
Soft Plaque Score, mean (SD)	7 (12)	1 (4)	12 (15)	2 (6)	12 (18)

## Data Availability

The clinical data generated and/or analyzed during the current study cannot be shared publicly due to patient privacy and the Privacy Act of Australia.

## Supplementary Materials

The following supporting information can be downloaded at: https://www.mdpi.com/article/10.3390/biom13060917/s1, Figure S1: The AUCs from bootstrapping tuning; Figure S2: Principle Component Analysis plot for the concentrations of 683 lipid species before and after normalization by batches; Figure S3: Scatter plots of predictive probability by LRS and FRS in two BMI sub-groups; Table S1: List of p-values from multi-variable linear regression models.
