# Supplementary material for: Lipidomics Profiling and Risk of Coronary Artery Disease in the BioHEART-CT Discovery Cohort
Source: Biomolecules. 2023 May 31;13(6):917. doi: 10.3390/biom13060917 (PMC10296370; doi:10.3390/biom13060917)
Supplement: Supplementary file 1 [file biomolecules-13-00917-s001.zip › biomolecules-2393513-supplementary.pdf]

Supplementary Data

Table S1. List of *p*-values from multi-variable linear regression models.

Informative variable	*t*-value (*p*-value)
BMI	1.367 (0.172)
Hypertension	0.055 (0.956)
Hypercholesterolaemia	−1.321 (0.187)

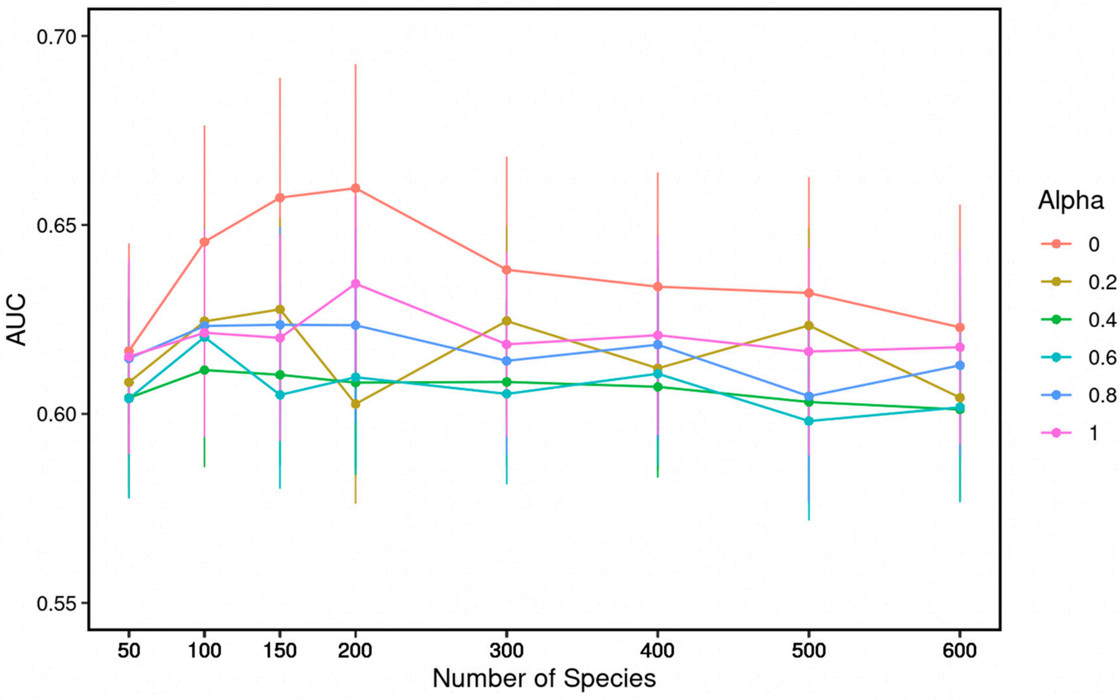

Figure S1. The AUCs from bootstrapping tuning. The range shows mean and 95% confidence intervals.

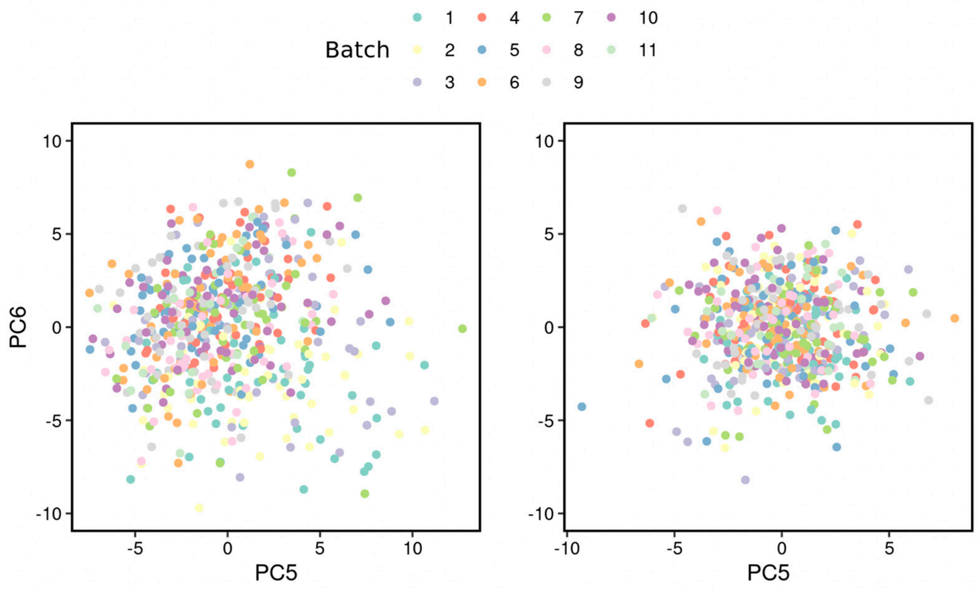

Figure S2. Principle Component Analysis plot for the concentrations of 683 lipid species before and after normalization by bacthes.

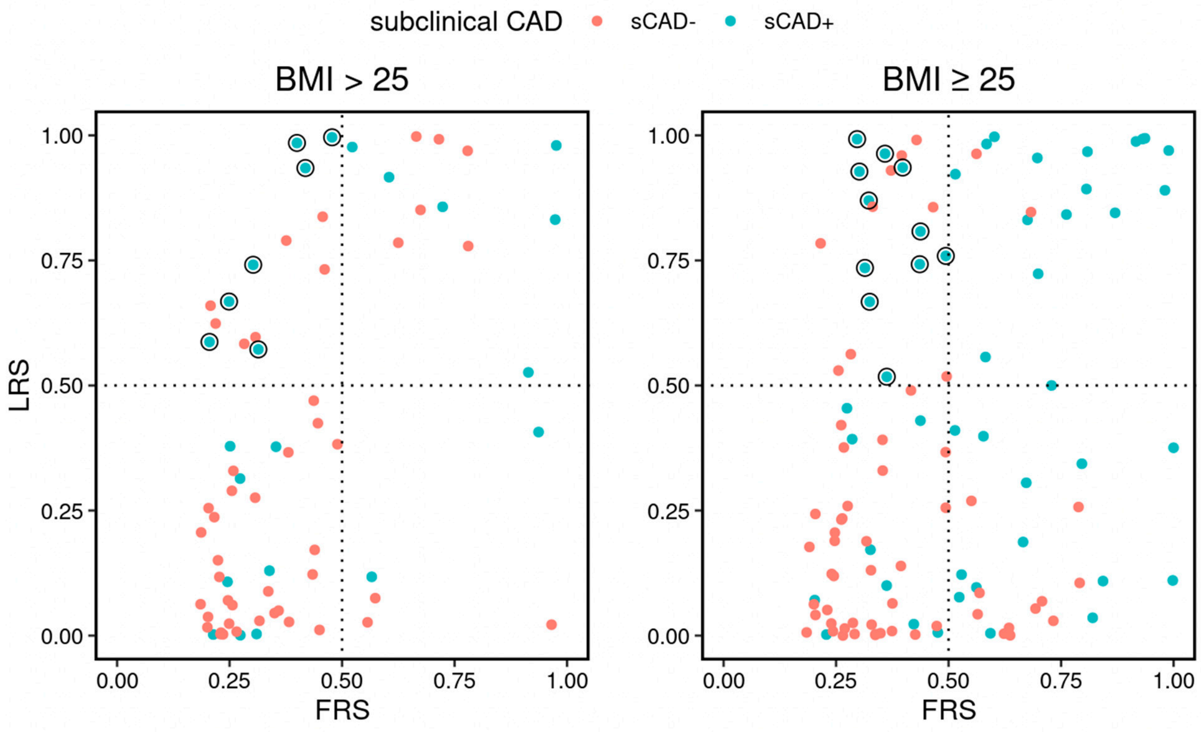

Figure S3. Scatter plots of predictive probability by LRS and FRS in two BMI sub-groups. The circles indicate individuals that can be precisely predicted by LRS but overlooked by the FRS model.
